# Computer‐Assisted Sperm Analysis (CASA) Versus Manual Evaluation and the Clinical Significance of Sperm Hyperactivated Motility

**DOI:** 10.1111/andr.70242

**Published:** 2026-04-21

**Authors:** Maria Emanuela Ragosta, Giulia Traini, Francesca Benini, Margherita Montereggi, Federica Bini, Lara Tamburrino, Linda Vignozzi, Elisabetta Baldi, Sara Marchiani

**Affiliations:** ^1^ Department of Experimental and Clinical Biomedical Sciences “Mario Serio” University of Florence Florence Italy; ^2^ IVIRMA Global Research Alliance, Demetra Florence Italy; ^3^ Andrology and Gender Endocrinology Unit, Center for Prevention, Diagnosis and Treatment of Infertility Careggi University Hospital Florence Italy; ^4^ Department of Experimental and Clinical Medicine University of Florence Florence Italy

**Keywords:** assisted reproduction, computer‐assisted sperm analysis, hyperactivated motility, ICSI, semen analysis, sperm count, sperm motility

## Abstract

**Background:**

Semen analysis remains the first‐line diagnostic test for male infertility, although it is affected by operator subjectivity. Computer‐assisted sperm analysis (CASA) is proposed to improve objectivity; however, its accuracy and clinical relevance remain uncertain.

**Objectives:**

We evaluated 424 samples from men attending routine semen analysis and 179 samples from male partners of ICSI couples to compare manual assessment with a CASA system (Ceros II), and to explore whether CASA‐derived kinematic parameters, in particular hyperactivated motility (HA), predict ICSI outcomes.

**Materials and Methods:**

Manual (according to WHO manual) and CASA assessments were compared using correlation analysis, Passing–Bablok regression, and Bland–Altman plots; kinematic parameters were evaluated in relation to ICSI outcomes.

**Results:**

Significant correlations were found between CASA and manual assessment for rapid progressive motility and for sperm count for samples with less than 50 × 10^6^/mL spermatozoa, whereas agreement was lost above this value, unless dilution was performed. Among CASA kinematic parameters, HA measured in whole semen was significantly higher in cycles achieving clinical pregnancy and resulted an independent pregnancy predictor also after adjustment for female age and female factor, showing good discriminative performance.

**Discussion:**

These findings suggest that CASA provides objective, reproducible assessment of sperm motility, and that hyperactivated motility may serve as a clinically relevant marker to improve prediction of ICSI outcomes.

**Conclusions:**

CASA may provide useful support for routine semen analysis and identify clinically meaningful kinematic parameters.

**Trial Registration:**

Not applicable

## 1 Introduction

In recent decades, the incidence of infertility among couples has risen significantly. This trend emphasized the importance of gaining a deeper understanding of the various factors affecting reproductive health. Although couple infertility is often linked with female issues, it also impacts men considerably, with male‐related factors contributing to approximately 50% of cases [1]. This highlights the need for a diagnostic approach that involves both partners, recognizing that male fertility plays a crucial role in the ability to conceive. Semen analysis provides detailed information on various sperm parameters, such as count, motility, viability, and morphology. These parameters collectively offer an overview of sperm quality and testicular function, although they do not represent a precise indicator of male fertility status [2]. Despite this limitation, semen analysis remains the first‐line test in the workup of male infertility. Therefore, it is crucial that the results of semen analysis are as accurate and precise as possible. To achieve this, every laboratory performing this analysis should apply the same methodology to ensure consistency and reliability of the results [3]. Such uniformity could be accomplished by following the manual for the examination and processing of human semen established by the World Health Organization (WHO), now in its sixth edition [4]. However, there are still concerns regarding the effective adoption of these standard procedures in all laboratories that perform semen analysis [5, 6]. Furthermore, not all laboratories participate in regular internal and external quality control programs. Another question concerns the comparability of results obtained by different technicians, as data can vary significantly depending on the skill and experience of the operator performing the analysis. This variability makes it challenging to compare results across different laboratories [7]. The need for rapid, objective, sensitive, and reliable analysis has led to one of the most significant innovations in semen analysis over the past 40 years: the development of computer‐assisted sperm analysis (CASA). The term CASA refers to an automated system, consisting of hardware and software, that captures and digitizes sequential images of spermatozoa. It processes and analyses these images to provide information on the kinematics of individual spermatozoa. The first commercial CASA systems were introduced in the 1980s, marking a significant step toward more precise and automated analysis. In recent years, these systems have undergone technological advancements in both hardware and software [8]. Specifically, modern CASA systems are no longer limited to kinematic parameters, but can also analyze advanced functional aspects, including hyperactivation, morphology, viability, DNA fragmentation, the ability to penetrate cervical mucus, and the acrosomal reaction [9].

Despite the implementations, the use of CASA in clinical routine is currently limited compared to its application in the veterinary field. Indeed, semen is likely the most challenging type of sample to analyze with a computer‐assisted image analysis, as it contains, in addition to spermatozoa, immature germ cells, seminal plasma, somatic cells, debris, and bacteria. Moreover, compared to other animal species, human semen can be highly viscous, spermatozoa are often aggregated, and there is significant variability in morphology. As a result, the main challenge of the system lies in distinguishing spermatozoa from other objects present in the whole semen sample [10], although the system is highly effective in analyzing selected spermatozoa, which typically have good motility and low/none contamination from other cells or debris. Many factors can therefore influence the performance of CASA systems, including sample preparation, its concentration, frame rate, the chamber used, and the optical conditions of the microscope system (particularly lighting and contrast). Despite continuous refinement of imaging technologies and improvements in analysis methods, the reliability of CASA systems still depends on the training, skill, and experience of the operator. In addition, given the variability of CASA instruments on the market and the different algorithms used, it is currently difficult to compare the results obtained from various systems.

This study had two main aims. The first was to compare semen analysis performed manually by experienced operators, according to the latest WHO manual [4], with that conducted using a CASA system (Ceros II, Hamilton Thorne, Beverly, MA, USA) by trained operators, in order to evaluate the agreement between the two methods in determining sperm motility and concentration. Specifically, we investigated whether CASA provides consistent results with manual assessment in both a population of men undergoing routine semen analysis for couple infertility and in a cohort of male partners of couples undergoing assisted reproductive technology (ART).

The second aim was to evaluate the clinical relevance of sperm kinematic parameters measured by CASA, both in whole semen and in spermatozoa selected for intracytoplasmic sperm injection (ICSI), by assessing their predictive value for ART outcomes. Together, these aims address both the methodological reliability of CASA across different patient populations and its potential clinical utility in assisted reproduction.

## 2 Materials and Methods

### 2.1 Study Population

Semen samples were consecutively collected from both 424 men undergoing routine semen analysis for couple infertility at the Andrology Laboratory of Careggi University Hospital, Florence, and 179 male partners of infertile couples undergoing ART cycles at the GENERALIFE Demetra Center, Florence. The study was approved by the local Ethics Committee (Ref. 20908_bio), and all participants provided written informed consent, including agreement that any remaining semen or selected spermatozoa could be used for research purposes following standard clinical ART procedures.

Among the ART couples, infertility causes were distributed as follows: 48.6% female factor, 5% male factor, 9% combined factors, and 37.4% idiopathic. Among female partners, 55.6% exhibited diminished ovarian reserve, 0.9% endometriosis, 4.6% polycystic ovary syndrome, and 38.9% other conditions. All couples underwent ICSI.

### 2.2 Semen Sample Collection and Manual Assessment

Semen samples from both cohorts were collected after 2–7 days of sexual abstinence through masturbation into a plastic container. Semen analysis was performed within 30–60 min after ejaculation according to the WHO [4] manual. After macroscopic evaluation of the sample, sperm motility was assessed using a phase‐contrast microscope (Leica DM LS; Leica), with 40× objective and a heated plate set at 37°C. The motility was determined by evaluating the percentage of at least 200 spermatozoa on two different slides and classifying the sperm motility as rapid‐progressive, slow‐progressive, non‐progressive, or immotile. Sperm viability was assessed using a 1% eosin stain, with at least 200 spermatozoa counted and discrimination made between non‐stained viable (white heads) and eosin‐stained nonviable (pink heads) cells. Sperm concentration was determined using the Improved Neubauer cell counting chamber after dilution of the sample in a formalin‐containing buffer. Sperm morphology was scored under an optical microscope with a 100× oil‐immersion objective, determining the percentage of normal and abnormal forms, after Diff‐Quik staining.

Both manual and CASA sperm analysis was performed by three highly trained technicians who have participated in the United Kingdom National External Quality Assessment Service (UK‐NEQAS) external quality control program for semen analysis since 2005. The mean (± SD) percent biases for the laboratory for the year 2024 were 3.2 (± 12.7) for progressive motility, 4.7 (± 11.8) for total motility, and of 3.4 (± 6.9) for a sperm concentration (*n* = 16, data from UK‐NEQAS). Inter‐operator (manual: 21.2 ± 4.3; CASA: 17.8 ± 5.7) and individual (manual: 3.2 ± 1.9; CASA: 1.4 ± 0.6) coefficients of variation (CVs) were calculated across representative samples for both manual and CASA analyses to assess reproducibility and agreement. For the ART cohort, semen analysis prior to sperm selection was performed by different operators.

Sperm selection for oocyte insemination was performed by swim‐up or density gradient centrifugation according to sample characteristics.

For swim‐up, samples were centrifuged at 300 × *g* for 10 min, the supernatant was removed, and 0.5 mL of Sperm Wash Medium supplemented with 1% HSA was layered over the pellet. After incubation at 37°C, the upper 0.5 mL fraction was recovered.

For density gradient centrifugation, samples were layered onto 45%/90% PureSperm gradients and centrifuged at 400 × *g* for 10 min, followed by a washing step at 300 × *g* for 10 min. The final pellet was resuspended in 0.5 mL of Sperm Wash Medium supplemented with 1% HSA. After selection, sperm count and motility were evaluated, and the selected fraction was used for insemination.

### 2.3 Computer‐Assisted Sperm Analysis

The CASA system (Ceros II, Hamilton Thorne, Beverly, MA, USA) includes three main components: an optical microscope (Olympus CX41 with negative phase‐contrast, dark‐field illumination, and a 37°C heated plate), a digital camera and a computer. The software version is HT CASA II version 1.6. Samples were placed into Leja slides with two chambers of 20 µm depths for analysis (Cryo Bio System, Groupe I.M.V. Technologies, Saint‐Ouen‐sur‐Iton, France). Acquisition was performed under 100× magnification. The settings used during the CASA procedure were those set by the system for human spermatozoa: analysis duration was 1 s (30 frames); the maximum and minimum head size was 50 and 5 µm^2^, respectively; minimum head brightness was set to 170; and minimum tail brightness was set to 70. For each sample, at least 200 motile spermatozoa were examined across a minimum of five fields, up to a maximum of 15 fields. Throughout the analysis, the samples were kept at a constant temperature of 37°C to ensure the reliability of the results [4].

The following kinematic parameters were recorded: average path velocity (VAP, µm/s), straight line velocity (VSL, µm/s), curvilinear velocity (VCL, µm/s), amplitude of lateral head displacement (ALH, µm), beat cross frequency (BCF, Hz), straightness (STR, %), and linearity of progression (LIN, %). According to Mortimer [11], the threshold values of VCL ≥ 150 µm/s, ALH ≥ 7 µm, and LIN ≤ 50% were set to identify a fraction representing the percentage of hyperactivated spermatozoa (HA, %).

For the first aim of the study, CASA analysis was performed on the same semen samples previously evaluated manually. Samples with concentrations higher than 50 million/mL were analyzed in three ways: (1) undiluted, (2) diluted 1:1 with seminal plasma, and (3) diluted 1:1 with physiological solution.

For the second aim, CASA was used to analyze both whole semen samples and corresponding selected sperm samples obtained by swim‐up or density gradient centrifugation. Semen samples were analyzed by performing manual and CASA assessments simultaneously. A small aliquot of each sample was used for CASA analysis, while the remaining portion was used for manual evaluation. Sperm motility was continuously monitored during the analysis to ensure it remained stable, minimizing any potential impact of timing on the results.

### 2.4 Ovarian Stimulation, ICSI, and Embryo Quality Evaluation

All patients underwent standard ovarian stimulation protocols individualized according to age, BMI, ovarian reserve, and prior response. Follicular growth was monitored by serum estradiol and ultrasound, and final oocyte maturation was triggered with hCG. Oocyte retrieval was performed ∼35 h later under sedation and local anesthesia. Mature oocytes (metaphase II) underwent ICSI using standard micromanipulation techniques, and fertilization was assessed 17 ± 1 h post injection by the presence of two pronuclei. Embryos were cultured to the blastocyst stage and graded according to Gardner criteria, which consider both blastocyst expansion (numerical score 1–6) and the quality of the inner cell mass and trophectoderm (letter grades A–C). Blastocysts were classified as high‐quality (1–6 AA, AB, BA, or BB) or low‐quality (1–6 BC, CB, CC, CD, DC, or DD), reflecting overall development and cellular organization. Surplus embryos were cryopreserved regardless of quality.

Single blastocyst transfer was performed on Day 5 post oocyte retrieval based on morphology. Pregnancy outcome was evaluated by β‐hCG (≥ 5 mIU/mL) and confirmed by ultrasound between Weeks 9 and 10. Cumulative pregnancy rates included both fresh and frozen embryo transfers, as no differences in ART outcomes were observed between them.

### 2.5 Statistical Analysis

Statistical analysis was performed using the Statistical Package for the Social Sciences version 29.0 (SPSS) for Windows. Since data were not normally distributed, as assessed by the Kolmogorov–Smirnov test, results are presented as medians and interquartile ranges (IQRs) or CVs. Semen parameters evaluated using manual and automated methods were compared using the Wilcoxon test and Spearman's rank correlation coefficient was calculated. Agreement between methods and the presence of systematic errors were evaluated using Bland–Altman plots and Passing–Bablok regression. The Bland–Altman analysis calculates the mean difference between results from the two methods to identify systematic bias. The Passing–Bablok regression is a non‐parametric method that makes no assumptions about sample distribution or measurement errors, and the result is not influenced by how the methods are assigned to X and Y. The slope and intercept are calculated with their 95% confidence intervals (CIs), and methods were considered comparable if the slope is within the 95% CI and the intercept is zero. In addition, an Andrews plot was generated to provide an exploratory multivariate visualization, to highlight possible differences between CASA and manual data. The plot was based on the following parameters: percentage of rapidly progressive motile spermatozoa, slowly progressive motile spermatozoa, total progressive spermatozoa, non‐progressive spermatozoa, immotile spermatozoa, and sperm concentration. Bland–Altman analysis, Passing–Bablok regression, and Andrews plot were generated using R Studio version 2024.12.0+467 (Boston, MA, USA).

Mann–Whitney *U*‐test was used to compare the following groups: fertilization rate (number of fertilized oocytes/numbers of inseminated oocytes) < 80% versus ≥ 80%; cleavage rate (number of embryos/number of fertilized oocytes) < 33% versus ≥ 33%; high versus low embryo quality, pregnant versus nonpregnant women. Binary logistic regression model was performed for multivariate analysis to adjust for confounding factors known to influence ART outcomes, such as female age [12, 13] and female factor of infertility [14, 15, 16].

Receiver operating characteristic (ROC) curve analysis was applied to determine diagnostic performance, expressed as area under the curve (AUC) with 95% CIs, as well as sensitivity and specificity for predicting the achievement of clinical pregnancy.

## 3 Results

### 3.1 Comparison Between Manual and Automated Semen Evaluation

**FIGURE 1:**
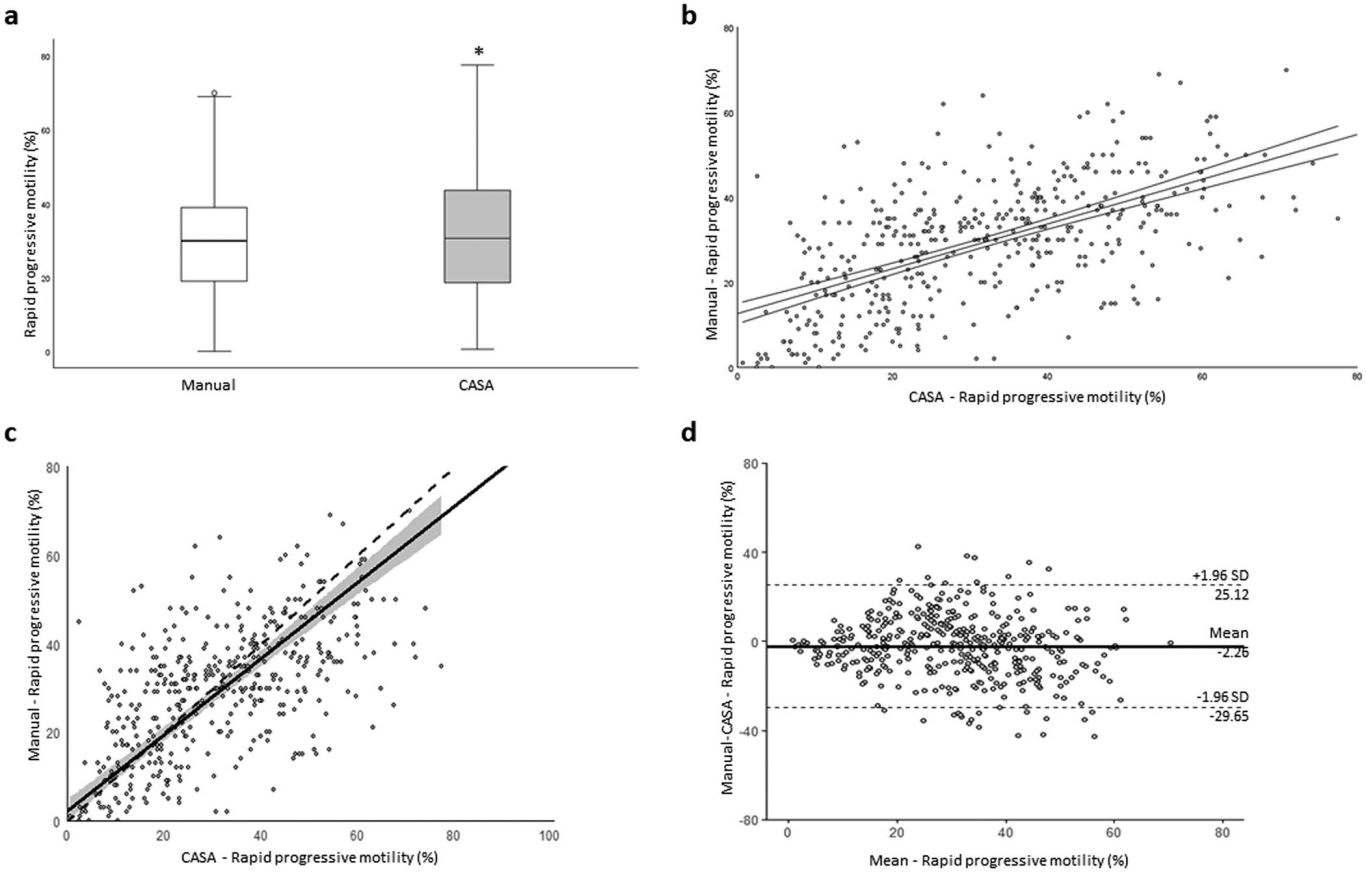
Comparison of rapid progressive sperm motility assessed by manual evaluation and CASA. (a) Boxplots showing the percentage of rapid progressive motility measured manually and by CASA (*n* = 424). *p < 0.05 versus manual. (b) Scatter plot illustrating the correlation between manual and CASA values. The solid line represents the linear regression line with its 95% confidence intervals (*R* = 0.6, *p* < 0.001; *n* = 424). (c) Passing–Bablok regression analysis comparing manual and CASA measurements. The regression equation (*y* = 2.41 + 0.86*x*) is shown along with the confidence interval band and the identity line (dashed). (d) Bland–Altman plot assessing agreement between manual and CASA measurements. The solid line indicates the mean difference between methods, and dashed lines represent the limits of agreement (± 1.96 SD).

A total of 424 male subjects were included in the study, with a median age of 36.0 years (IQR: 30.0–41.0) and a median period of sexual abstinence of 4.0 days (IQR: 3.0–5.0). Rapid progressive spermatozoa showed comparable values between the two methods, with a median of 30.0% (IQR: 19.0–39.0) for manual assessment and a slightly higher median of 30.6% (IQR: 18.6–43.7, *p* < 0.05, Figure 1a) for CASA. A moderate positive correlation was observed (*R* = 0.6, *p* < 0.001, *n* = 424, Figure 1b), and regression analysis using the Passing–Bablok method yielded the equation *y* = 2.41 + 0.86*x* (95% CI for the slope: 0.78–0.93; intercept: −0.001 to 4.95), suggesting a proportional but not perfect alignment (Figure 1c). The Bland–Altman plot confirmed a moderate agreement, with a mean difference of −2.26% (manual–CASA) and wide limits of agreement (−29.65% to 25.12%), indicating a slight underestimation by the manual method (Figure 1d).

**FIGURE 2:**
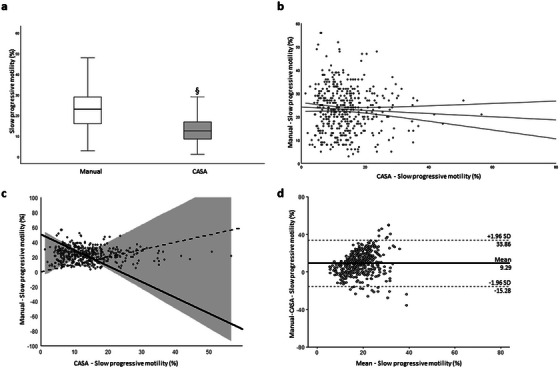
Comparison of slow progressive sperm motility assessed by manual evaluation and CASA. (a) Boxplots showing the percentage of slow progressive motility measured manually and by CASA (*n* = 424). §*p* < 0.001 versus manual. (b) Scatter plot illustrating the correlation between manual and CASA values. The solid line represents the linear regression line with its 95% confidence intervals (*R* = −0.1, *p* = ns; *n* = 424). (c) Passing–Bablok regression analysis comparing manual and CASA measurements. The regression equation (*y* = 50.74−2.14*x*) is shown along with the confidence interval band and the identity line (dashed). (d) Bland–Altman plot assessing agreement between manual and CASA measurements. The solid line indicates the mean difference between methods, and dashed lines represent the limits of agreement (± 1.96 SD).

**FIGURE 3:**
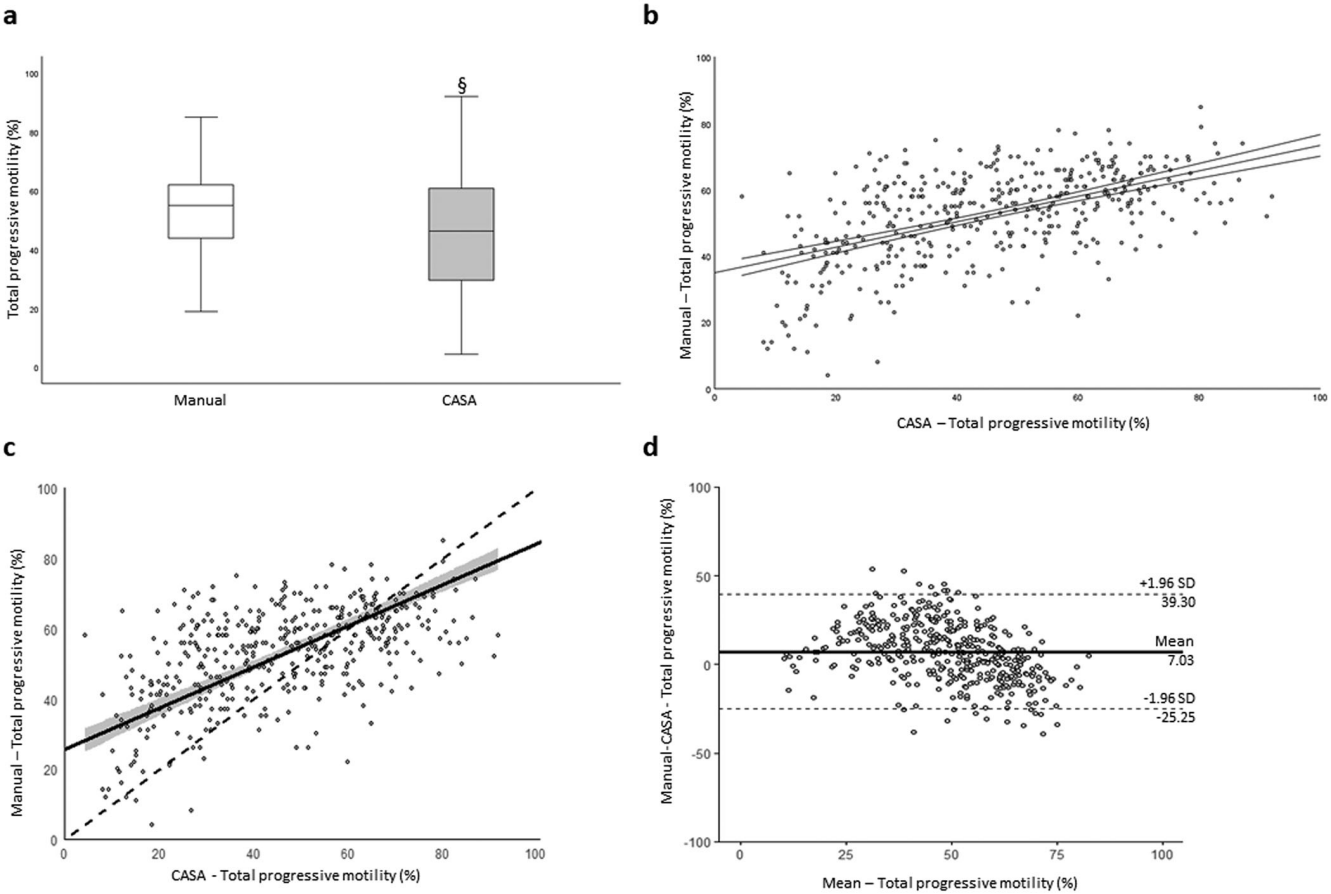
Comparison of total progressive sperm motility assessed by manual evaluation and CASA. (a) Boxplots showing the percentage of total progressive motility measured manually and by CASA (*n* = 424). §*p* < 0.001 versus manual. (b) Scatter plot illustrating the correlation between manual and CASA values. The solid line represents the linear regression line with its 95% confidence intervals (*R* = 0.5, *p* < 0.001; *n* = 424). (c) Passing–Bablok regression analysis comparing manual and CASA measurements. The regression equation (*y* = 25.84 + 0.59*x*) is shown along with the confidence interval band and the identity line (dashed). (d) Bland–Altman plot assessing agreement between manual and CASA measurements. The solid line indicates the mean difference between methods, and dashed lines represent the limits of agreement (± 1.96 SD).

Slowly progressive spermatozoa revealed marked discrepancies between the methods. Manual analysis yielded a median of 23.0% (IQR: 16.0–29.0), almost double the CASA value of 12.5% (IQR: 8.6–16.9; *p* < 0.001; Figure 2a). Correlation analysis showed a weak and non‐significant negative association (*R* = −0.1, *p* = ns, *n* = 424; Figure 2b), while Passing–Bablok regression (*y* = 50.74 − 2.14*x*) and its wide §CIs (slope: −2.59 to 2.35) suggested no consistent trend (Figure 2c). The Bland–Altman analysis showed a mean difference of 9.29% (limits ranging from −15.28% to 33.86%; Figure 2d). These results indicate that CASA systematically underestimates this parameter, and the two methods cannot be considered interchangeable. When examining total progressive spermatozoa (rapid + slow), the manual method again produced higher values (median 55.0%, IQR: 44.0–62.0) than CASA (median 46.3%, IQR: 29.6–60.9; *p* < 0.001; Figure 3a). A moderate correlation was evident (*R* = 0.5, *p* < 0.001, *n* = 424; Figure 3b), and regression analysis (Passing–Bablok equation: *y* = 25.24 + 0.59*x*) confirmed a systematic difference (Figure 3c), with the CASA system consistently yielding lower measurements. Bland–Altman analysis showed a mean difference of 7.03%, with broad limits (−25.25% to 39.30%; Figure 3d). Despite a moderate level of agreement, the methods showed variability, with the manual method tending to overestimate values.

**FIGURE 4:**
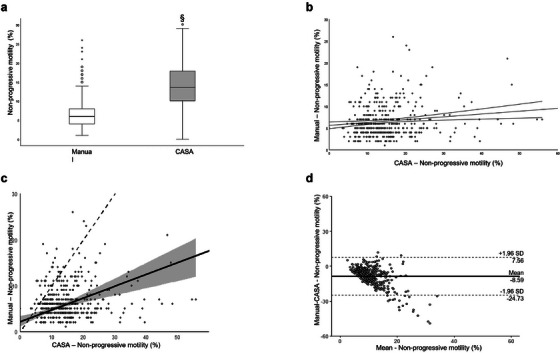
Comparison of non‐progressive sperm motility assessed by manual evaluation and CASA. (a) Boxplots showing the percentage of non‐progressive motility measured manually and by CASA (*n* = 424). §*p* < 0.001 versus manual. (b) Scatter plot illustrating the correlation between manual and CASA values. The solid line represents the linear regression line with its 95% confidence intervals (*R* = 0.1, *p* < 0.05; *n* = 424). (c) Passing–Bablok regression analysis comparing manual and CASA measurements. The regression equation (*y* = 2.16 + 0.26*x*) is shown along with the confidence interval band and the identity line (dashed). (d) Bland–Altman plot assessing agreement between manual and CASA measurements. The solid line indicates the mean difference between methods, and dashed lines represent the limits of agreement (± 1.96 SD).

Non‐progressive spermatozoa showed a reversed trend. CASA values were substantially higher (median 13.6%, IQR: 10.1–17.9) compared to the manual assessment (median 6.0%, IQR: 4.0–8.0; *p* < 0.001) (Figure 4a). Although statistically significant, the correlation was low (*R* = 0.1, *p* < 0.05, *n* = 424; Figure 4b), and the Passing–Bablok regression equation (*y* = 2.16 + 0.26*x*) revealed only a weak proportional association (Figure 4c). Bland–Altman analysis demonstrated a mean difference of −8.59%, with limits from −24.73% to 7.56% (Figure 4d). These findings point to a consistent overestimation by CASA. For immotile spermatozoa, both evaluations provided closely aligned results. The manual method yielded a median of 39.0% (IQR: 32.0–48.0), slightly higher than CASA (median 37.8%, IQR: 24.0–54.7; *p* < 0.05). Correlation was moderate (*R* = 0.5, *p* < 0.001, *n* = 424), and regression analysis indicated a consistent proportional relationship (*y* = 21.76 + 0.49*x*, 95% CI for slope: 0.43–0.55). However, the Bland–Altman plot showed considerable variability, with a mean difference of 1.62% and wide limits (−32.01% to 35.26%; data not shown).

**FIGURE 5:**
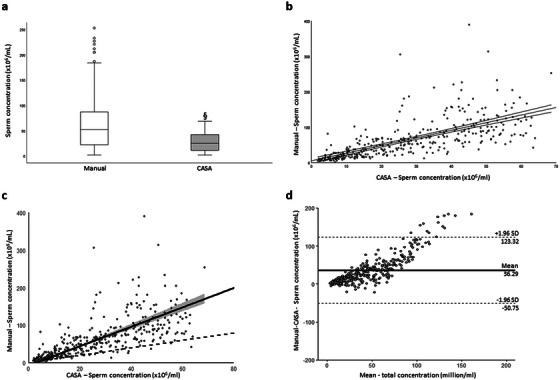
Comparison of sperm concentration (× 10^6^/mL) assessed by manual evaluation and CASA. (a) Boxplots showing sperm concentration (× 10^6^/mL) measured manually and by CASA (*n* = 373). §*p* < 0.001 versus manual. (b) Scatter plot illustrating the correlation between manual and CASA values. The solid line represents the linear regression line with its 95% confidence intervals (*R* = 0.7, *p* < 0.001; *n* = 373). (c) Passing–Bablok regression analysis comparing manual and CASA measurements. The regression equation (*y* = −7.55 + 2.59*x*) is shown along with the confidence interval band and the identity line (dashed). (d) Bland–Altman plot assessing agreement between manual and CASA measurements. The solid line indicates the mean difference between methods, and dashed lines represent the limits of agreement (± 1.96 SD).

Sperm concentration analysis, performed in 373 samples, revealed the most pronounced divergence. Manual counts were significantly higher (median: 52.0 × 10^6^/mL, IQR: 22.0–87.0) than CASA values (median: 25.1 × 10^6^/mL, IQR: 11.0–42.2; *p* < 0.001; Figure 5a). A strong positive correlation was found (*R* = 0.7, *p* < 0.001, *n* = 373; Figure 5b), but Passing–Bablok regression (*y* = −7.55 + 2.59*x*) indicated that CASA tended to increasingly underestimate concentrations as values rose (Figure 5c). Bland–Altman analysis confirmed this tendency with a large mean difference of 36.29% and very wide limits of agreement (−50.75% to 123.32%; Figure 5d).

To better understand this divergence, we stratified the samples into two groups: those with concentrations ≥ 50 × 10^6^/mL (*n* = 191) and those with < 50 × 10^6^/mL (*n* = 184; Figure S1). In both groups, the manual method continued to report significantly higher values than CASA (*p* < 0.001). For samples ≥ 50 × 10^6^/mL, the median manual concentration was 101.5 × 10^6^/mL (IQR: 67.0–122.0) versus 39.7 × 10^6^/mL by CASA (IQR: 28.3–50.4). For the < 50 × 10^6^/mL group, median values were 22.0 × 10^6^/mL (IQR: 13.0–34.0) and 11.0 × 10^6^/mL (IQR: 6.9–19.1), respectively. Correlation analysis revealed a moderate relationship in the high‐concentration group (*R* = 0.3, *p* < 0.001, *n* = 191), while a stronger correlation was seen in the lower concentration group (*R* = 0.6, *p* < 0.001, *n* = 184). Passing–Bablok regression reflected the same pattern: for samples ≥ 50 × 10^6^/mL, the regression equation was *y* = −53.86 + 3.80*x* (95% CI for slope: 2.93–5.24; Figure S1a); for samples < 50 × 10^6^/mL, the equation was *y* = 1.73 + 1.69*x* (95% CI for slope: 1.40–1.98; Figure S1c). Bland–Altman analysis further confirmed better agreement at lower concentrations, with a mean difference of 9.75% (limits: −10.52% to 30.01%) for the < 50 × 10^6^/mL group (Figure S1d), compared to 61.82% (limits: −34.62% to 158.25%) for the ≥ 50 × 10^6^/mL group (Figure S1b).

Given the notable discrepancies between the two methods for samples with high sperm concentrations, we investigated whether dilution could enhance the accuracy of CASA measurements. Within the ≥ 50 × 10^6^/mL group, three conditions were explored: undiluted samples, samples diluted 1:1 with physiological saline, and samples diluted 1:1 with seminal plasma. Results demonstrated a clear impact of dilution on CASA performance. Dilution with physiological saline improved the agreement, resulting in a moderate correlation (*R* = 0.5, *p* < 0.001, *n* = 139; Figure S2a), and a Passing–Bablok regression equation of *y* = 44.80 + 0.93*x* (Figure S2b). After dilution in seminal plasma the correlation become higher (*R* = 0.7, *p* < 0.001, *n* = 57; Figure S2a) with a regression equation of *y* = 24.55 + 1.63*x* (Figure S2c). Bland–Altman analysis revealed substantial variability of the obtained values: for saline dilution, the mean bias was 35.75% (limits: −75.11% to 146.62%; Figure S2b); for seminal plasma dilution, the mean bias was 48.5% (limits: −12.67% to 109.67%; Figure S2c).

An Andrews plot was generated for the 424 semen samples to explore potential multivariate separation between CASA and manual assessments. The resulting plot showed largely overlapping data, indicating that, despite some statistically significant differences, the two methods produce highly similar overall patterns (Figure S3).

### 3.2 Comparison of Manual and CASA Assessment in the Cohort of Male Partners of Couples Undergoing ART

To verify whether the discrepancies observed in the main cohort of 424 men were also present in clinical ART setting, total progressive motility was evaluated in a cohort of 179 male partners of couples undergoing ART for which manual assessment was performed by different trained operators on the same day of IVF procedure (see M&M). In this cohort, pre‐selection total progressive motility showed comparable median values between the two methods, with 42.0% (IQR: 30.0–55.0) for manual assessment and 41.8% (IQR: 27.9–58.6) for CASA. Correlation revealed only a moderate association (*R* = 0.48, *p* < 0.001; data not shown). Passing–Bablok regression (*y* = 12.44 + 0.65*x*) indicated a proportional relationship with a slight tendency of CASA to underestimate higher motility values, while Bland–Altman analysis confirmed fair agreement (mean difference −2.21%, limits −38.38% to 34.0%; data not shown).

### 3.3 Predictive Value of Sperm Kinematic Parameters for ART Outcomes

**FIGURE 6:**
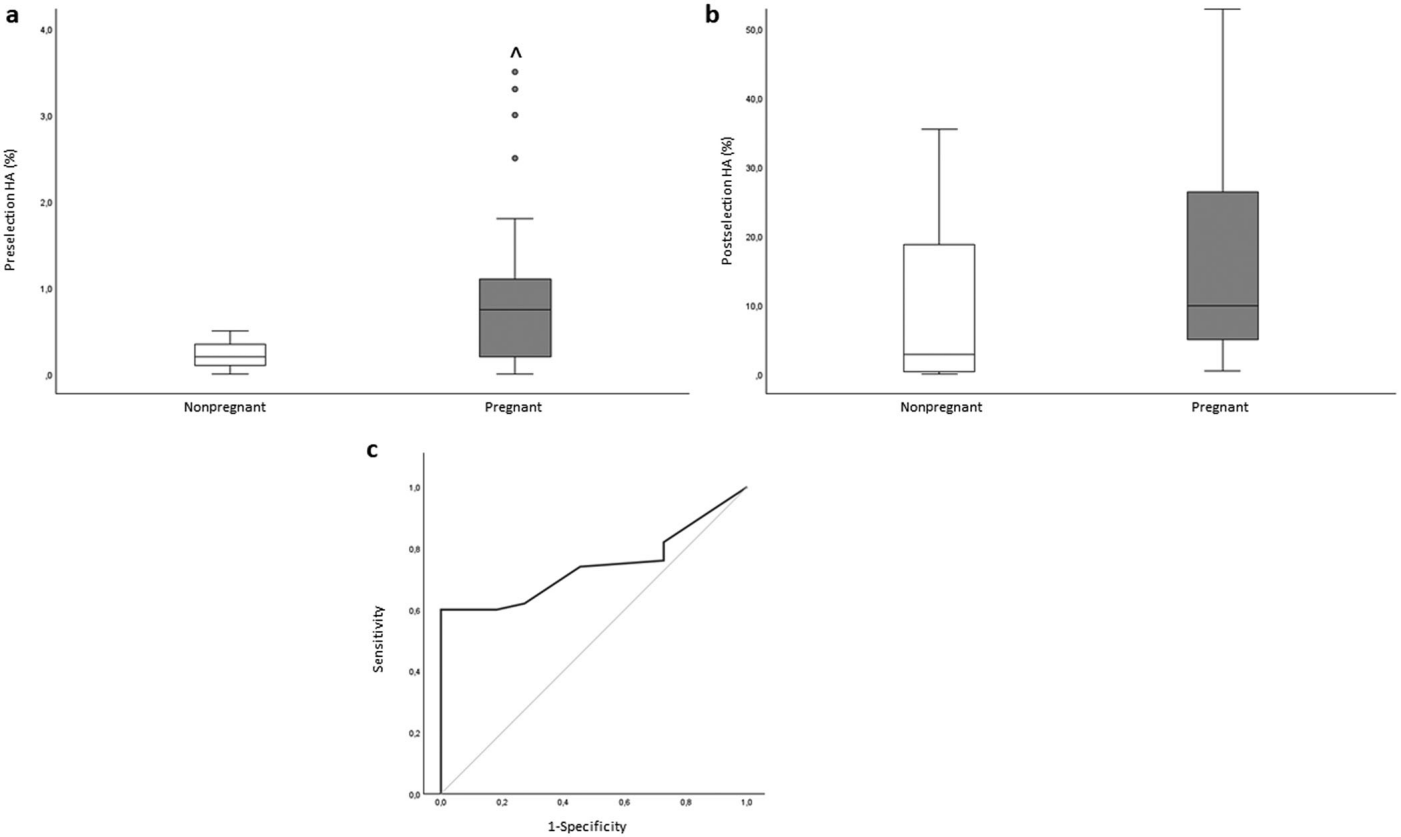
Box plots showing the percentage of hyperactivated sperm motility (HA) in the pregnant and non‐pregnant groups before (a) and after sperm selection (b), ^p<0.02. (c) Receiver operating characteristic (ROC) curve illustrating the predictive ability of pre‐selection HA for achieving a clinical pregnancy.

No significant differences were found in any kinematic parameters between groups for early ART outcomes (fertilization rate, cleavage rate, high vs. low embryo quality), either before or after sperm selection (data not shown). Interestingly, HA evaluated before selection was significantly higher in the group achieving clinical pregnancy compared with the non‐pregnant group (Figure 6a). For post‐selection HA, a similar trend was observed, although statistical significance was not reached (Figure 6b). The same pattern was seen for rapid progressive motility, total progressive motility, VAP, VCL, and VSL, all of which showed higher values in the pregnancy group, but without reaching significance (data not shown).

A binary logistic regression model was applied to adjust for potential confounding factors, including female age and the presence of a female infertility factor. Pre‐selection HA remained a significant predictor of clinical pregnancy (*p* = 0.019), indicating that higher HA values were associated with an approximately 14‐fold increased probability of achieving pregnancy. ROC curve analysis yielded an AUC of 0.741 (95% CI: 0.618–0.864; *p* < 0.001). An HA threshold of 0.65 provided a specificity of 100% and a sensitivity of 60% (Figure 6c).

## 4 Discussion

Accurate assessment of semen quality is fundamental for both the diagnosis of male infertility and the optimization of ART. While manual semen analysis remains the gold standard, it is subject to interoperator variability [5, 6], is time‐consuming, and requires skilled operators, thereby motivating the increasing use of CASA systems in recent years. These systems can accurately measure several key parameters, including sperm motility, concentration, morphology, viability, and kinetics, analyzing a large number of cells in a shorter time compared to manual method [17]. However, despite its potential, CASA performance is dependent on sample quality and the algorithms used for sperm identification. Early versions of these systems were limited by operational constraints, often resulting in inaccurate tracking and compromising the reproducibility and comparability of data across different laboratories and studies.

Our study provides a detailed evaluation of CASA performance across different semen parameters and patient populations, and explores the clinical relevance of kinematic parameters, particularly HA, in predicting ART outcomes. Notably, in our knowledge, this represents the largest study to date comparing manual semen analysis and CASA, including the highest number of subjects in which differences between these two methods have been evaluated, thus strengthening the robustness of our findings. Nonetheless, the monocentric design and the use of a single CASA platform and software version represent limitations.

Our results indicate that CASA provides generally reliable measurements for several semen parameters, although its performance varies depending on the specific parameter considered. Rapid progressive spermatozoa showed moderate correlation and minor differences between manual and CASA measurements, suggesting that CASA effectively captures this type of motility respect to human eye. Since rapid progressive motility is the most important predictor of sperm fertilizing potential [18, 19], accurate evaluation by the CASA system of this parameter highlights its potential relevance for clinical decision‐making and ART outcomes. In contrast, slowly progressive spermatozoa were consistently underestimated by CASA, with weak correlations and poor agreement, likely due to limitations in accurately distinguishing slow progressive from nonprogressive spermatozoa. Indeed, CASA tended to overestimate non‐progressive motility, further supporting the presence of frequent misclassification and reduced concordance with manual evaluation. However, slow progressive motility per se did not show significant associations with reproductive outcomes in our cohort, supporting the concept that the fraction of highly motile spermatozoa may be biologically more relevant. Total progressive motility reflected these trends, with CASA reporting lower values than manual assessment, although a moderate correlation is found. Evaluation of total progressive motility in the ART cohort confirmed trends observed in the main population, with moderate correlation and fair agreement between methods. Notably, manual motility assessment during ART procedures could be less precise than in routine semen analysis, even if performed by trained operators. In this context, operators generally perform a rapid, overall evaluation aimed at determining the sperm selection strategy and the most appropriate ART procedure, rather than conducting a detailed sperm assessment for precise diagnostic purposes. Inter‐operator variability may have also contributed to the observed differences. This pragmatic approach may partially explain the differences observed between manual and CASA measurements in this cohort. Overall, while median values were closely aligned, agreement between the two methods was slightly lower, and variability was higher in the ART cohort.

In the main population, CASA also yielded acceptable values for immotile spermatozoa. In our analysis, this parameter would appear reliable, as our version of the instrument is probably less susceptible to a common limitation of CASA systems, that is, the potential misclassification of debris with size similar to sperm heads as immotile cells. The CASA system used in this study operates with negative phase‐contrast microscopy, the standard configuration for this platform. However, the presence of debris, non‐sperm cells, or other particles, particularly in raw semen samples, may interfere with automated recognition algorithms, potentially leading to misclassification and inaccuracies in sperm count or motility assessment [17]. Therefore, each acquired field was systematically verified to ensure that non‐sperm particles were not erroneously recorded as spermatozoa.

In general, the observed discrepancies likely reflect differences in how motility is assessed. Manual evaluation is, by nature, subjective, as even highly trained operators may interpret sperm motility subtypes differently, resulting in variability in the measurements [20, 21]. In contrast, CASA relies on predefined algorithms to classify sperm movement, which standardizes the assessment but makes it dependent on the specific instrument and software version. Although newer CASA systems allow operators to review and reclassify sperm motility subtypes after image acquisition, this process requires trained personnel and increases analysis time. In the system used in the present study, the partial post‐acquisition correction, combined with intrinsic variability between manual and CASA analyses and the differing numbers of spermatozoa evaluated by each method, may have contributed to discrepancies.

Sperm concentration showed the most pronounced discrepancies. CASA provides reliable concentration measurements in samples with < 50 million/mL, but accuracy declines at higher concentrations unless proper dilution is performed, as confirmed by our experiments. This highlights the importance of standardized pre‐analytical protocols, particularly for high‐concentrated samples. Strategies such as increasing the number of analyzed fields or using complementary automated systems may help improve measurement reliability. Nevertheless, very low sperm concentrations remain technically challenging for some CASA systems and should be interpreted with caution.

Although Bland–Altman and Passing–Bablok analyses revealed some discrepancies between CASA and manual assessments, the Andrews plot indicates that the overall patterns largely overlap, suggesting that these differences may have limited biological significance. A recent systematic review by Finelli et al. [22] analyzed 14 studies comparing manual semen analysis and CASA. The review highlighted considerable discrepancies across studies: some reported a high level of agreement between manual and CASA measurements, whereas others observed significant differences. Importantly, the studies included in the review used different CASA instruments, varying in both brand and software version, which likely contributed to the heterogeneity of results. The review also emphasized that CASA results show high variability in samples with very low (< 15 million/mL) or very high (> 60 million/mL) sperm concentrations, reinforcing the need for careful standardization and strict adherence to protocols when using CASA in clinical practice. Moreover, the review concluded that further improvements in CASA technology are necessary, and robust validation studies are essential to establish the accuracy and precision of CASA measurements before human operators can be replaced. Our study, analyzing a robust caseload, supports these conclusions. We found that CASA systems can be quite reliable for evaluating rapid progressive motility and, consistent with the systematic review, that assessment of sperm concentration still requires careful attention in samples with very high sperm counts.

Beyond methodological evaluation, CASA provided clinically meaningful information. HA measured by CASA before sperm selection emerged as a significant predictor of clinical pregnancy, with higher HA values associated with increased probability of achieving pregnancy. This association remained significant even after adjusting for confounding factors, including female age and the presence of female infertility factors. ROC curve analysis further supported the predictive value of pre‐selection HA, with an AUC indicating fair discrimination between cycles resulting in clinical pregnancy versus non‐pregnancy. These findings highlight the potential utility of CASA‐derived kinematic parameters in supporting clinical decision‐making and improving ART outcomes, offering an advantage compared to conventional manual assessment, which is limited in its ability to capture the complex and dynamic movements of individual spermatozoa. This observation aligns with previous literature. Exploratory analyses of kinematic subpopulations, including rapid and slow progressive spermatozoa and subgroups defined by higher VAP and STR values, did not reveal additional significant associations with reproductive outcomes in our cohort (data not shown). While more advanced clustering approaches may provide further biological insight, the present findings suggest that HA represents the most informative kinematic parameter in this context.

A key limitation of our study is that all ART cycles were performed using ICSI rather than conventional IVF. Because ICSI involves the direct microinjection of spermatozoa into oocytes, bypassing many natural steps required for fertilization, this may explain the lack of association we observed between HA and fertilization, as well as other early outcomes, in contrast to previous IVF‐based studies [23, 24]. Nonetheless, our data suggest that HA may still identify a sperm subpopulation with superior kinematic and functional characteristics, potentially conferring reproductive advantage even in ICSI cycles. Notably, a significant association with clinical pregnancy was observed only before sperm selection, whereas post‐selection HA showed a similar trend without reaching statistical significance. Although some variability was present before selection, HA values were generally higher after selection, however, contrary to expectations, variability increased rather than decreased (see Figure 6a,b). This increased dispersion suggests that sperm selection may amplify underlying biological differences between samples, possibly reflecting heterogeneity in sperm responsiveness to selection procedures, thereby partially masking associations with clinical outcomes.

Clinical translation of HA addresses methodological challenges. External validation in independent cohorts is essential before routine clinical implementation. A major limitation is the lack of universally accepted cut‐off for defining HA by CASA, together with inter‐laboratory variability in instrument settings and algorithms, which further complicate standardization. Differences in kinematic parameter thresholds can strongly influence the percentage of spermatozoa classified as hyperactivated, limiting comparability across studies and laboratories.

In conclusion, CASA provides a reliable and efficient alternative to manual semen analysis for many key parameters, in particular rapid progressive motility and low–moderate sperm concentrations. Limitations remain for slow progressive motility and high‐concentration samples, but ongoing optimization and implementation are likely to improve reliability across all parameters.

In addition, CASA can quantify kinematic parameters such as HA, which in our study predicted clinical pregnancy even after adjusting for confounding factors. Although the predictive value of HA may be attenuated in ICSI cycles, it still appears to identify samples with enhanced functional characteristics which may contribute to improve reproductive outcomes.

Overall, CASA reduces operator‐dependent variability and provides clinically meaningful information that complements conventional semen analysis. Further multicenter studies with standardized protocols and HA definitions are urgently needed to validate these findings and optimize clinical application of CASA systems in ART.

## Author Contributions

*Conceptualization*: Sara Marchiani and Elisabetta Baldi. *Methodology*: Sara Marchiani, Francesca Benini, and Lara Tamburrino. *software*: Maria Emanuela Ragosta. *validation*: Sara Marchiani and Elisabetta Baldi. *Formal analysis*: Maria Emanuela Ragosta, Margherita Montereggi, Federica Bini, and Giulia Traini. *Investigation*: Maria Emanuela Ragosta and Giulia Traini. *Data curation*: Maria Emanuela Ragosta. *Writing – original draft preparation*: Maria Emanuela Ragosta. *Writing – review and editing*: Sara Marchiani. *Supervision*: Sara Marchiani, Elisabetta Baldi, and Linda Vignozzi. *Funding acquisition*: Linda Vignozzi and Sara Marchiani. All the authors have read and agreed to the published version of the manuscript.

## Funding

This research was funded by University of Florence to Sara Marchiani, the European Union—NextGenerationEU‐National Recovery and Resilience Plan, Mission 4 Component 2‐Investment 1.5‐THE‐Tuscany Health Ecosystem‐ECS00000017‐CUP B83C22003920001 to Linda Vignozzi, and the Italian Ministry of University (MUR, project 381 PRIN‐PNRR P2022FA79R) to Sara Marchiani.

## Ethics Statement

The study was conducted in accordance with the Declaration of Helsinki, and approved by the Local Ethics Committee (Ref. 20908_bio, date of approval 01.03.2022) for studies involving humans.

## Consent

Informed consent was obtained from all subjects involved in the study.

## Conflicts of Interest

The authors declare no conflicts of interest.

## Supporting information




**SupportingFile 1**: andr70242‐sup‐0001‐SuppMat.docx.

## Data Availability

The data that support the findings of this study are available upon request from the corresponding author. The data are not publicly available due to privacy or ethical restrictions.
