# Supplementary material for: Computer‐Assisted Sperm Analysis (CASA) Versus Manual Evaluation and the Clinical Significance of Sperm Hyperactivated Motility
Source: Andrology. 2026 Apr 21;14(5):1401–12. doi: 10.1111/andr.70242 (PMC13266430; doi:10.1111/andr.70242)
Supplement: Supplementary file 1 — SupportingFile 1: andr70242‐sup‐0001‐SuppMat.docx. [file ANDR-14-1401-s001.docx]

**COMPUTER ASSISTED SPERM ANALYSIS (CASA) VERSUS MANUAL EVALUATION AND THE CLINICAL SIGNIFICANCE OF SPERM HYPERACTIVATED MOTILITY**

Authors: Maria Emanuela Ragosta ^1^, Giulia Traini ^1^, Francesca Benini ^2^, Margherita Montereggi ^2^, Federica Bini ^2^, Lara Tamburrino ^3^, Linda Vignozzi ^1,3^, Elisabetta Baldi ^4^ and Sara Marchiani ^1,3^ .

**Institutions**: ^1^Department of Experimental and Clinical Biomedical Sciences "Mario Serio", University of Florence, 50134 Florence, Italy;

^2^ IVIRMA Global Research Alliance, Demetra, Florence, Italy;

^3^ Andrology and Gender Endocrinology Unit, Center for Prevention, Diagnosis and Treatment of Infertility, Careggi University Hospital, 50139 Florence, Italy;

^4^ Department of Experimental and Clinical Medicine, University of Florence, 50134 Florence, Italy.

**Supplementary Figure legends:**

**
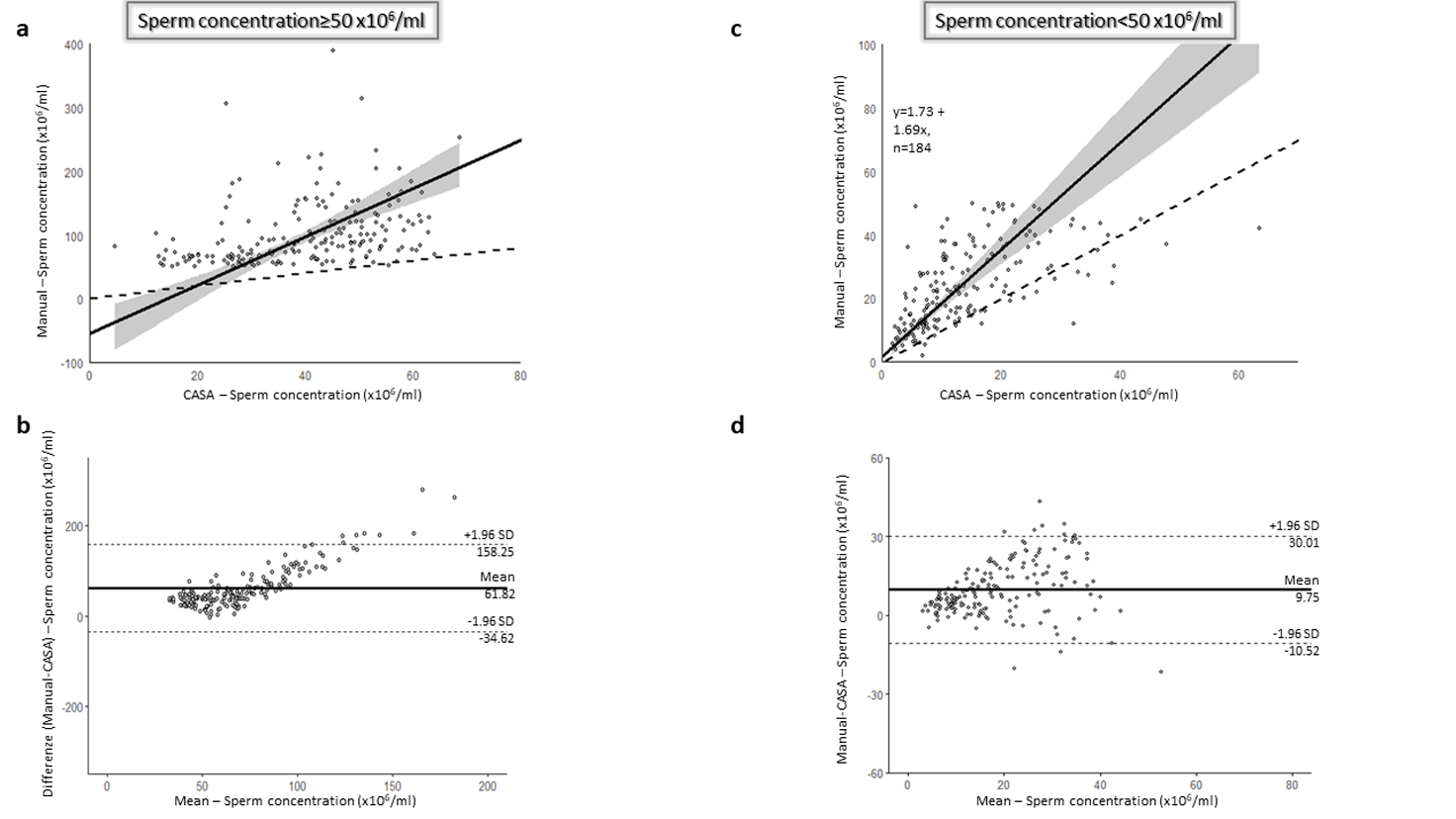
**

**Figure S1.** Stratification of semen samples based on sperm concentration (≥50x10^6^/ml, n=191; <50x10^6^/ml, n=184). (**a**) and (**c**) Passing–Bablok regression analyses comparing manual and CASA sperm concentration measurements in the two groups. The regression equations (y=-53.86+3.8x for the ≥50x10^6^/ml group; y=1.73+1.69x for the <50x10^6^/ml group) are shown along with the confidence interval band and the identity line (dashed). (**b**) and (**d**) Bland–Altman plots assessing agreement between manual and CASA measurements in the two groups. The solid line indicates the mean difference between methods, and dashed lines represent the limits of agreement (±1.96 SD).

**
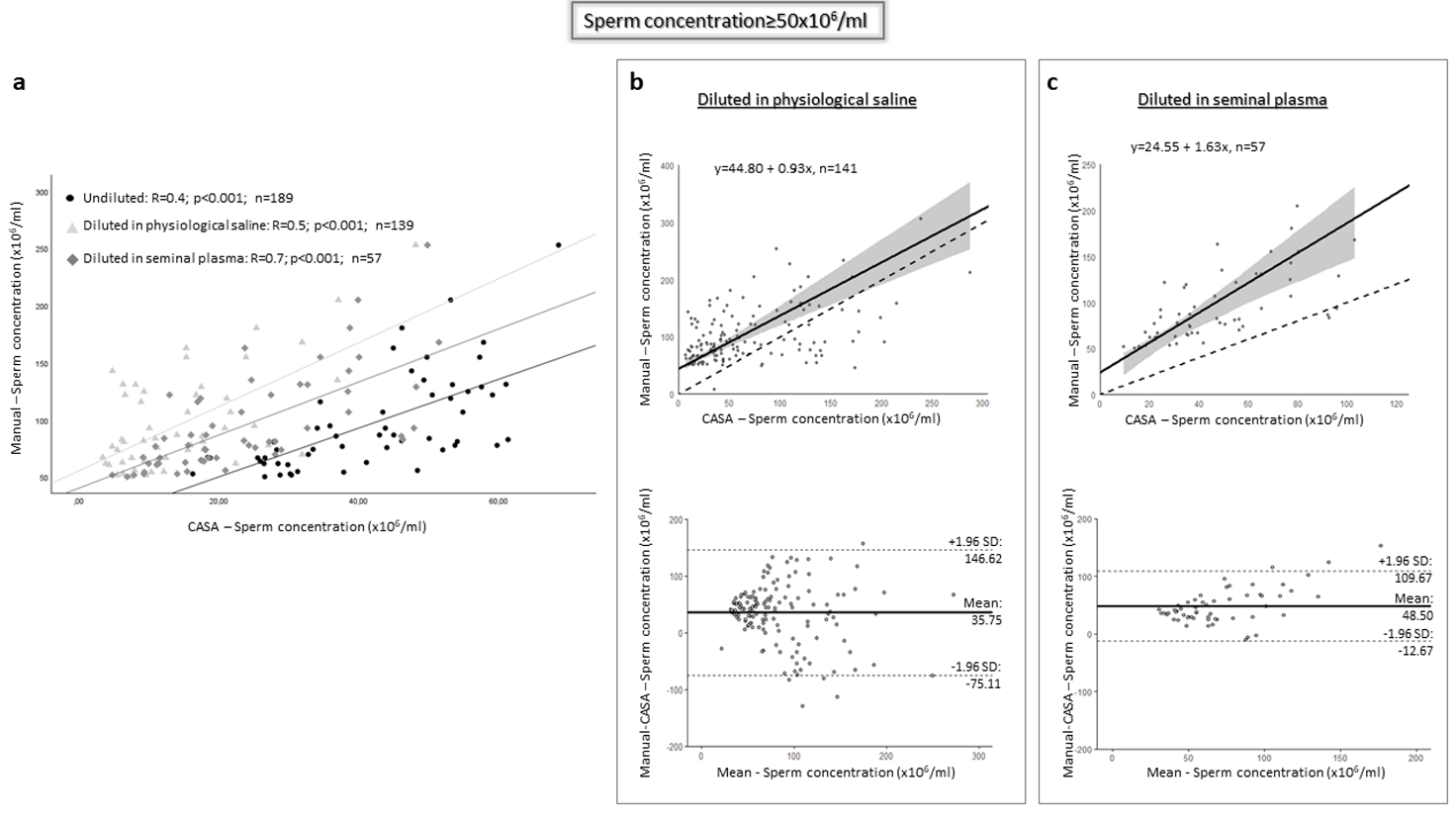
**

**Figure S2.** (**a**) Scatter plot illustrating the correlation between manual and CASA sperm concentration (×10⁶/ml) in semen samples with concentrations ≥50 ×10⁶/ml under three conditions: undiluted samples (black circles), samples diluted 1:1 with physiological saline (light grey triangles), and samples diluted 1:1 with seminal plasma (dark grey diamonds). Solid lines represent the linear regression lines. (**b**) and (**c**), upper panels: Passing–Bablok regression analyses comparing manual and CASA measurements in samples diluted with physiological saline and seminal plasma, respectively. The regression equations (y = 44.80 + 0.93x for physiological saline; y = 24.55 + 1.63x for seminal plasma) are shown together with the confidence interval bands and the identity line (dashed). (**b**) and (**c**), lower panels: Bland–Altman plots assessing the agreement between manual and CASA measurements in samples diluted with physiological saline and seminal plasma, respectively. The solid line indicates the mean difference between methods, while dashed lines represent the limits of agreement (±1.96 SD).

**
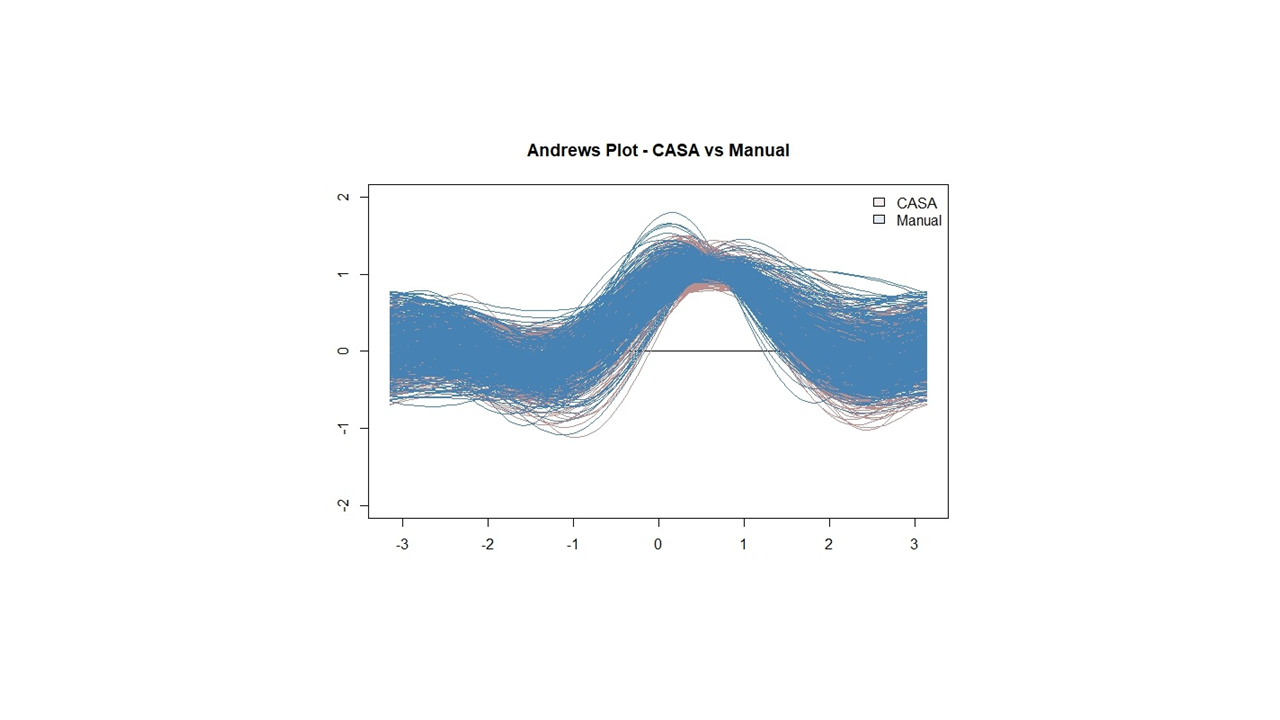
Figure S3**. Andrews plot comparing CASA (dusty rose) and manual evaluation (steel blue) of 424 sperm samples, based on rapidly progressive, slowly progressive, totally progressive, non-progressive, and immotile sperm percentages, as well as sperm concentration (millions/mL).
